# Global bibliometric analysis of traditional Chinese medicine regulating gut microbiota in the treatment of diabetes from 2004 to 2024

**DOI:** 10.3389/fphar.2025.1533984

**Published:** 2025-01-23

**Authors:** Jieling Liang, Xiaojuan Lin, Xin Liao, Xi Chen, Ying Zhou, Lin Zhang, Yunyun Qin, Haoru Meng, Zhongwen Feng

**Affiliations:** ^1^ Department of Pharmacy, Guilin Hospital of the Second Xiangya Hospital, Central South University, Guilin, China; ^2^ Department of Pharmacy, Guangxi Academy of Medical Sciences and the People’s Hospital of Guangxi Zhuang Autonomous Region, Nanning, China; ^3^ Phase 1 Clinical Trial Laboratory, Guangxi Academy of Medical Sciences and the People’s Hospital of Guangxi Zhuang Autonomous Region, Nanning, China

**Keywords:** traditional Chinese medicine, diabetes, gut microbiota, active constituents, mechanism

## Abstract

**Objectives:**

The therapeutic efficacy of Traditional Chinese Medicine (TCM) in modulating gut microbiota for diabetes treatment has garnered increasing scholarly attention. This study aims to meticulously examine current research trajectories and focal areas from 2004 to 2024, providing a foundational framework for future inquiries.

**Methods:**

A comprehensive search of documents published between 2004 and 2024 was conducted using the Web of Science database. The resulting data were analyzed and visualized using R software, VOSviewer, and CiteSpace.

**Results:**

The study included a total of 751 documents. From 2004 to 2022, the number of annual publications showed a continuous upward trend (2004: n = 1 to 2022: n = 159), and the number of publications in 2023 (n = 141) decreased slightly from the previous year. China emerged as the leading country in terms of article publications (n = 430). Additionally, the United States played a prominent role in international research collaborations. Frontiers in Pharmacology (n = 31) was the most frequently published journal, while Nature (n = 1,147) achieved the highest citation count. Key identified keywords included obesity, insulin resistance, inflammation, and oxidative stress.

**Conclusion:**

Three key research focuses in this domain include: the therapeutic effects of active constituents in TCM on diabetes via gut microbiota modulation, the underlying mechanisms through which TCM influences gut microbiota in diabetes management, and the targeted regulation of specific gut bacterial populations by TCM in the treatment of diabetes.

## 1 Introduction

Diabetes is a metabolic disorder characterized by chronic hyperglycemia, primarily encompassing type 1 diabetes mellitus (T1DM) and type 2 diabetes mellitus (T2DM), with the latter accounting for over 85% of diabetes cases (Patil et al., 2023). With global economic development and lifestyle changes, the prevalence of diabetes has rapidly increased (Abdul Basith Khan et al., 2020). According to the International Diabetes Federation, approximately 463 million adults worldwide had diabetes in 2019, and this number is expected to rise to 700 million by 2045 (Saeedi et al., 2019). Diabetes and its complications not only severely impact patients’ quality of life but also impose a significant economic burden on global healthcare systems. Currently, diabetes treatment mainly relies on insulin injections, oral hypoglycemic agents, and lifestyle interventions (Sivakumar et al., 2021; Prasathkumar et al., 2022). However, these traditional treatment methods have certain limitations. While insulin injections are effective, they suffer from poor patient compliance and can cause adverse reactions such as hypoglycemia (Cernea and Raz, 2020). Oral hypoglycemic agents, including metformin, sulfonylureas, and DPP-4 inhibitors, can effectively control blood glucose levels but may lead to side effects such as gastrointestinal discomfort and weight gain with long-term use (Ou et al., 2015; Yu et al., 2020). Therefore, exploring new methods for diabetes treatment that are safe, effective, and have minimal side effects has become an urgent need.

In recent years, the gut microbiota, as an important regulatory system of the internal environment, has garnered widespread attention in the pathogenesis and treatment of diabetes (Afzaal et al., 2022). The gut microbiota refers to the community of microorganisms residing in the human gut, playing crucial roles in host metabolism, immune regulation, nutrient absorption, and vitamin synthesis (Gomaa, 2020; Dey, 2024). Studies have found that the diversity of gut microbiota is significantly reduced in diabetic patients, characterized by a decrease in beneficial bacteria and an increase in harmful bacteria, a dysbiosis closely associated with the development and progression of diabetes (Sechovcová et al., 2024). For instance, the imbalance in the ratio of *Firmicutes to Bacteroidetes* in the gut of diabetic patients is closely linked to insulin resistance and low-grade chronic inflammation (Bajinka et al., 2023). Regulating the gut microbiota to restore its balance may provide new strategies for the prevention and treatment of diabetes.

Interestingly, many TCM and formulations have shown unique advantages in regulating gut microbiota and improving metabolic disorders. For example, berberine, a major active component of *Coptis chinensis*, improves insulin sensitivity and glucose metabolism by modulating gut microbiota composition and increasing the production of short-chain fatty acids (SCFAs) (Yang et al., 2023; Wang et al., 2023). Puerarin, found in *Pueraria lobata*, has also been discovered to alleviate insulin resistance by regulating gut microbiota (Xu et al., 2021a). Additionally, significant progress has been made in studying the regulatory effects of TCM formulations on gut microbiota. Classic prescriptions such as Huanglian Jiedu Decoction and Shenling Baizhu Powder not only regulate gut microbiota balance but also improve gut barrier function and reduce intestinal toxin levels, thereby exerting comprehensive anti-diabetic effects (Du et al., 2020; Zheng et al., 2020; Zhang et al., 2022a).

Despite these advancements, the vast body of literature on TCM, gut microbiota, and diabetes remains fragmented and lacks a systematic understanding of the research trends, key contributors, and knowledge gaps. Bibliometric analysis provides a quantitative approach to addressing these challenges, enabling the identification of research hotspots, collaboration networks, and emerging trends over time. However, a thorough bibliometric study specifically focused on the intersection of TCM, gut microbiota, and diabetes is still lacking. Existing bibliometric studies have primarily explored diabetes treatment strategies or gut microbiota research in isolation, leaving a critical gap in understanding the integrative role of TCM in gut microbiota modulation for diabetes management. This study aims to fill this gap by conducting a comprehensive bibliometric analysis, offering valuable insights into the development and potential future directions of this interdisciplinary field.

This study aims to systematically summarize and analyze the latest research progress on TCM and formulations regulating gut microbiota for diabetes treatment, exploring their specific mechanisms of action, clinical efficacy, and future research directions. Through bibliometric methods, we hope to provide a solid theoretical foundation for the scientific application of TCM in diabetes treatment and offer new ideas and methods for future related research.

## 2 Materials and methods

### 2.1 Data collection

The dataset was obtained from the Web of Science Core Collection (WoSCC) (Guangxi Medical University Purchase Edition) on 13 June 2024. The search strategy employed is detailed in Table 1. The retrieved documents were saved in plain text format and exported as complete records, inclusive of cited references. Following this, any extraneous or redundant entries were removed from the dataset.

**TABLE 1:** The research retrieval formula.

Step	Search expression	Results
#1	[TS = (“Chinese medicine” OR “traditional Chinese medicine” OR TCM OR “herbal medicine” OR “material medicine” OR decoction OR powder OR pill OR extract OR “Chinese proprietary medicine”)] AND TS = (diabetes)	27,281
#2	[TS = (gut OR bowel OR colon OR intestine* OR gastrointestin* OR gastro-intestine*)] AND TS = (“micro bio*” OR microbiome* OR microbiota OR microflora OR flora OR bacteria* OR commensal OR probiotic OR prebiotic OR microecology OR dysbiosis OR pathobiont)	161,831
#3	[(#1 AND #2) AND PY = (2004–2024) AND DT = (Article OR Review)] AND LA = (English)	751

### 2.2 Data analysis

To fully exploit the information contained within the downloaded data, we employed software such as the *Bibliometrix* package (version 4.0, http://www.bibliometrix.org) (Aria and Cuccurullo, 2017) within the R software environment (version 3.6.3), VOSviewer (version 1.6.17) (Van Eck and Waltman, 2010) and Citespace (version 6.2.4) (Chen, 2006) for analysis. These tools enabled the visual analysis of data and the generation of scientific knowledge maps. To ensure the accuracy and reliability of the data, two independent authors conducted the data extraction and analysis separately. Impact Factors (IF) for journals were obtained from the 2023 Journal Citation Reports (JCR).

VOSviewer was employed to generate visualizations of national collaboration networks, source co-citation analysis, and keyword co-occurrence. The criteria for co-authorship network analysis required a minimum of 5 documents per country and a minimum of 5 documents per organization. For source co-citation analysis, a source needed at least 200 citations. Additionally, for keyword co-occurrence analysis, a keyword had to appear at least 10 times, excluding those terms used in the search query and their synonyms, as their high frequency would obscure the analysis of other potentially interesting keywords. The description of the software used in the research is provided in Supplementary Annex 1.

## 3 Results

### 3.1 Overview of selected studies

**FIGURE 1:**
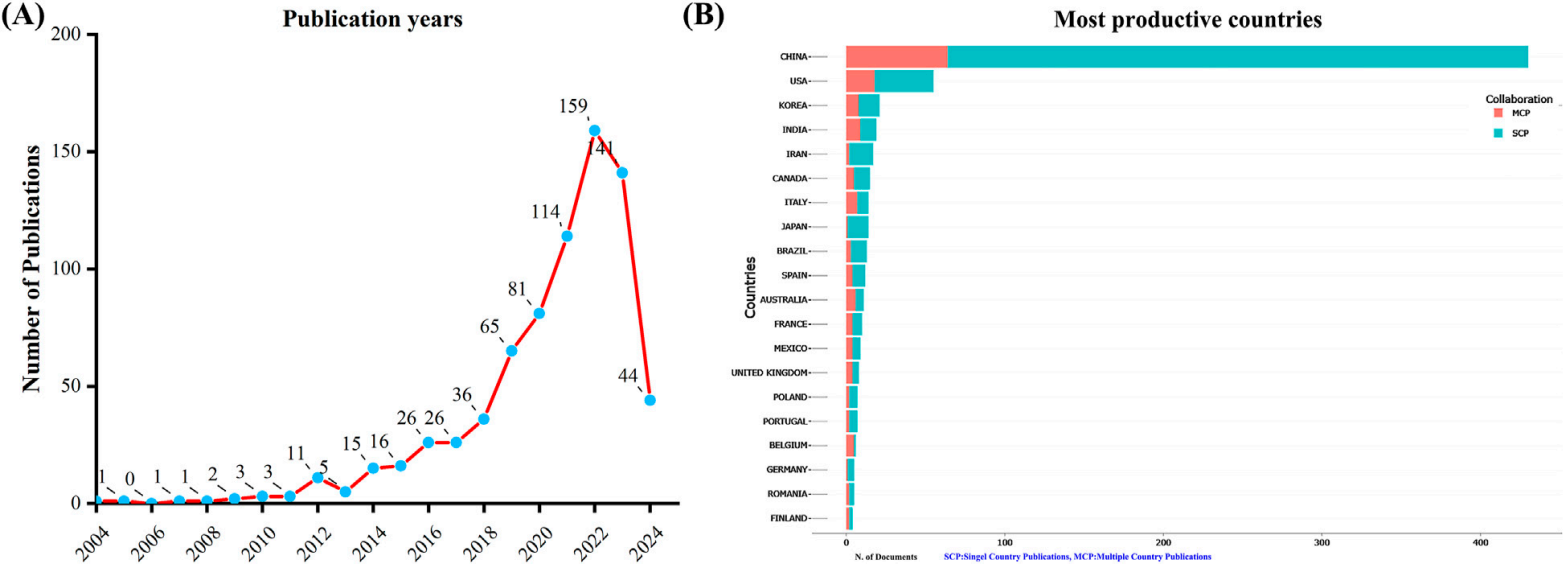
Annual publication trends in the field of TCM regulating gut microbiota in the treatment of diabetes from 2004 to 2024. **(A)** Depicts the annual publication trends. **(B)** Illustrates the distribution of countries and the collaborative efforts among corresponding authors.

A total of 751 unique records were collected from the WoSCC after removing duplicates. From 2004 to 2022, the number of documents related to TCM treatment for diabetes through gut microbiota has shown a consistent upward trend. However, there were fewer publications in 2023 than in 2022 (Figure 1A). This suggests that the field of study may be starting to get less attention.

**FIGURE 2:**
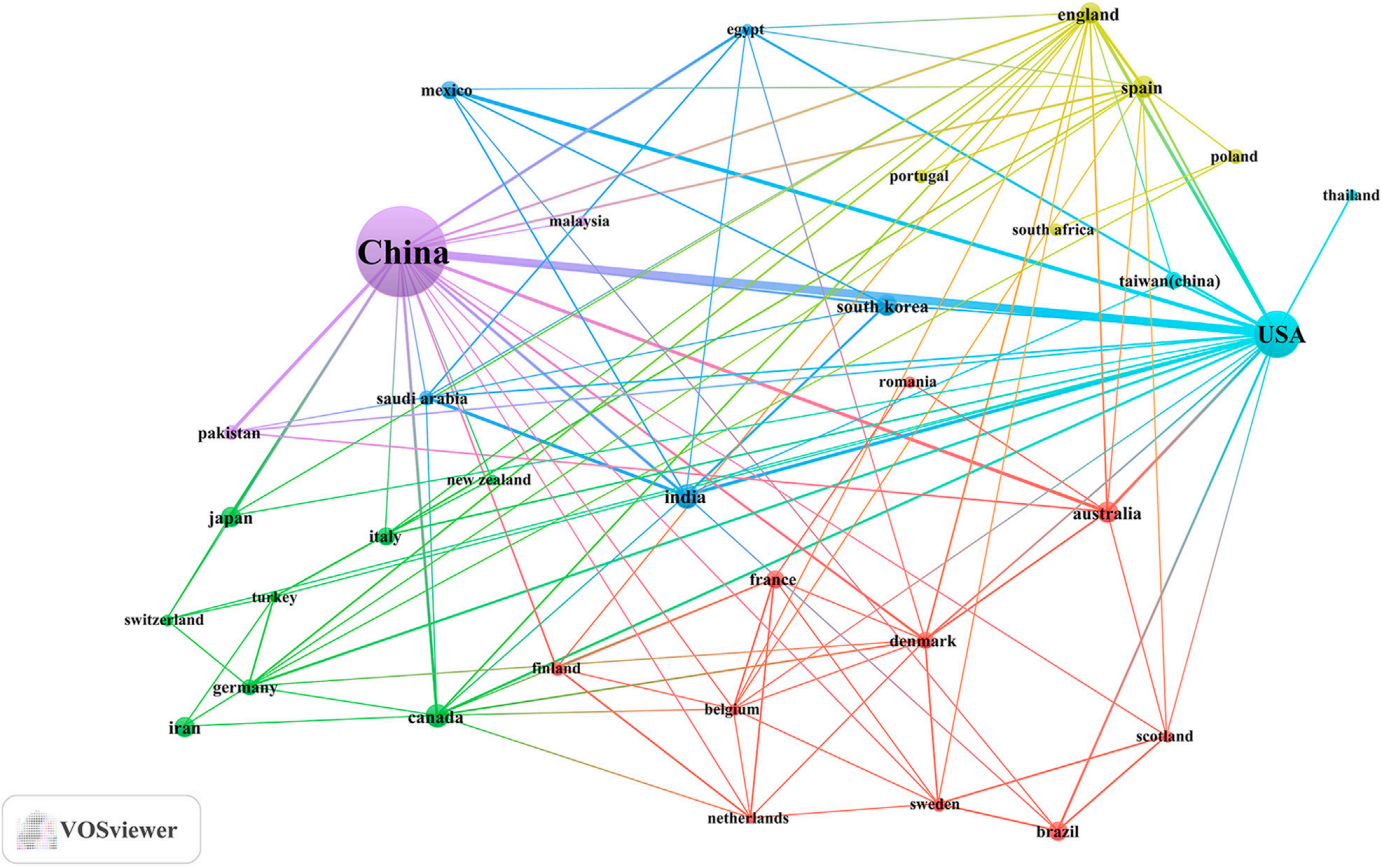
The map of countries involved in the field of TCM regulating gut microbiota in the treatment of diabetes from 2004 to 2024.

An analysis of the corresponding authors’ countries revealed that China (n = 430) was the leading publisher, followed by the United States (n = 55), Korea (n = 21), India (n = 19), and Iran (n = 17). Moreover, only 14.90% of publications from China involved multi-country collaborations (MCPs), a figure lower than the United States’s 32.7%, as illustrated in Figure 1B and detailed in Table 2. Notably, while China leads in publication volume, the United States maintains a more extensive network of international partnerships, as shown in Figure 2. This underscores the need for Chinese researchers to prioritize international collaboration and to approach the study of TCM treatment for diabetes through gut microbiota from a global perspective.

**TABLE 2:** Most relevant countries by corresponding authors.

Country	Articles	SCP	MCP	Freq	MCP_Ratio
China	430	366	64	0.573	0.149
United States	55	37	18	0.073	0.327
Korea	21	13	8	0.028	0.381
India	19	10	9	0.025	0.474
Iran	17	15	2	0.023	0.118
Canada	15	10	5	0.02	0.333
Italy	14	7	7	0.019	0.5
Japan	14	13	1	0.019	0.071
Brazil	13	10	3	0.017	0.231
Spain	12	8	4	0.016	0.333
Australia	11	5	6	0.015	0.545
France	10	6	4	0.013	0.4
Mexico	9	5	4	0.012	0.444
United Kingdom	8	4	4	0.011	0.5
Poland	7	5	2	0.009	0.286
Portugal	7	5	2	0.009	0.286
Belgium	6	1	5	0.008	0.833
Germany	5	4	1	0.007	0.2
Romania	5	3	2	0.007	0.4
Finland	4	2	2	0.005	0.5

Note: MCP, multiple country publication; SCP, single country publication.

### 3.2 Journal analysis and visualization

To explore the journals making significant contributions in the field of TCM treatment for diabetes through gut microbiota, we utilized the *Bibliometrix* package in R software. Graphical representations were generated using the ggplot2 package. Additionally, co-cited journal analysis was performed using VOSviewer.

**FIGURE 3:**
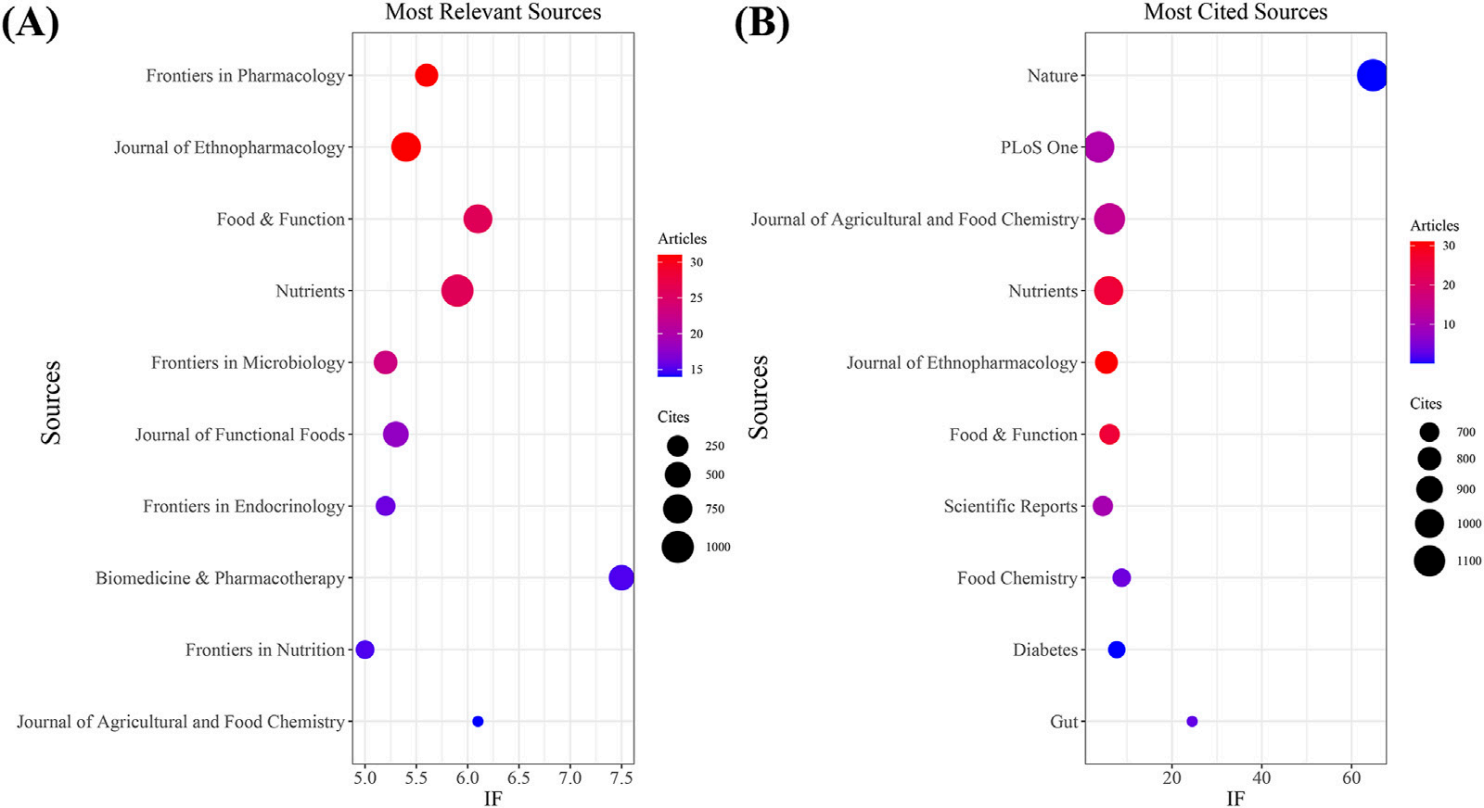
The journal with the highest volume of published articles and the journal with the most extensive citation count. **(A)** The journal with the highest quantity of published documents. **(B)** The journals with the most substantial citation counts.

**FIGURE 4:**
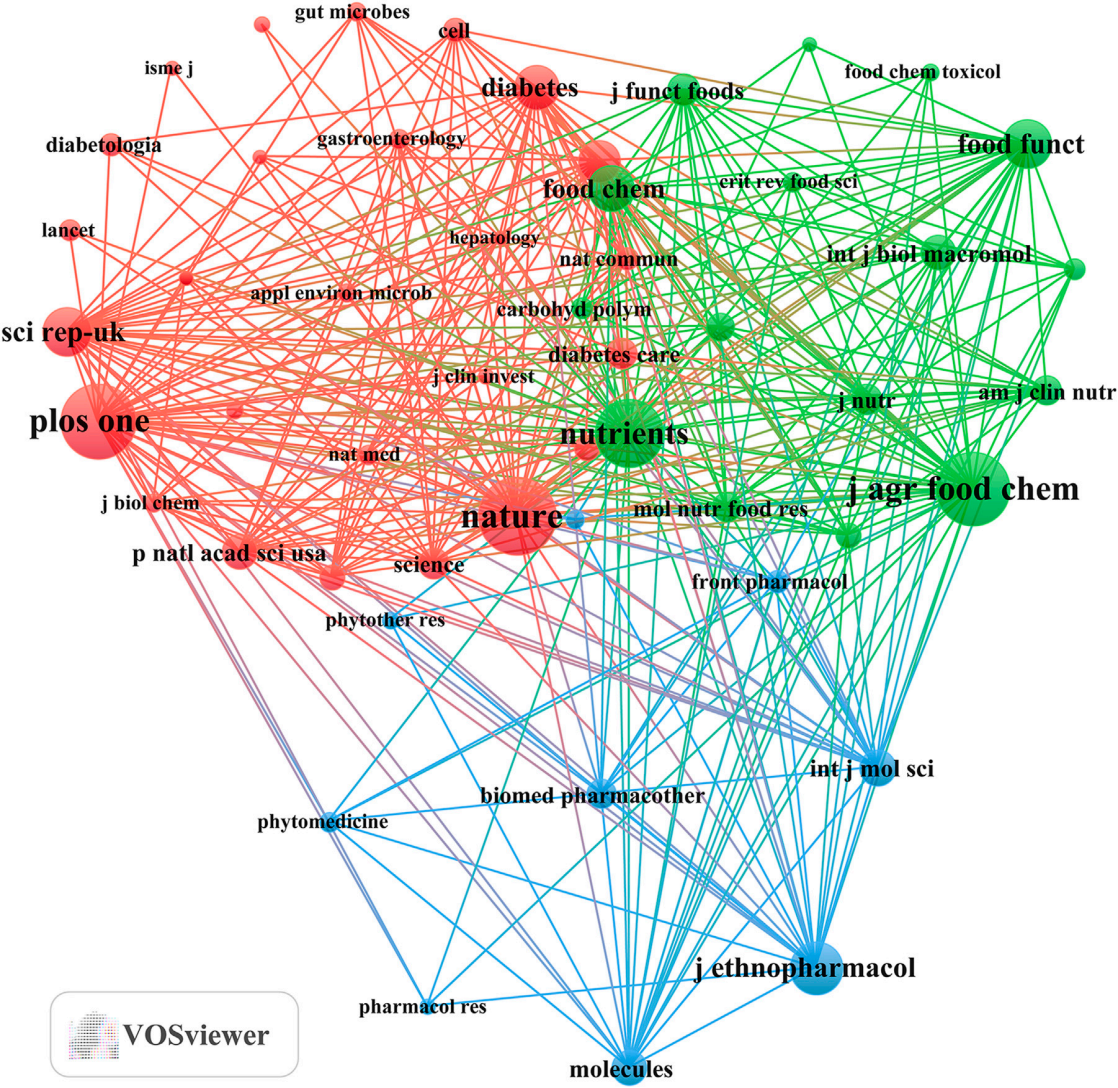
Co-cited journals involved in TCM regulating gut microbiota in the treatment of diabetes.

Our investigation identified a total of 751 documents distributed across 288 scholarly journals (detailed information in Supplementary Annex 2). As shown in Table 3 and depicted in Figure 3A, *Frontiers in Pharmacology* (n = 31, IF = 5.6) and *Journal of Ethnopharmacology* (n = 31, IF = 5.4) emerged as the top publishers, followed by *Food and Function* (n = 26, IF = 6.1), *Nutrients* (n = 26, IF = 5.9), and *Frontiers in Microbiology* (n = 23, IF = 5.2). Table 4; Figure 3B highlight the most frequently cited journals, including *Nature* (n = 1,147, IF = 64.8), *PLoS One* (n = 1,095, IF = 3.7), *Journal of Agricultural and Food Chemistry* (n = 1,087, IF = 6.1), Nutrients (n = 1,000, IF = 5.9), and *Journal of Ethnopharmacology* (n = 2,779, IF = 5.4). Importantly, the co-cited journals map in Figure 4 reveals that *Nature*, *Journal of Ethnopharmacology*, and *Nutrients* serve as central collaboration hubs. These findings collectively underscore the influential role of the *Journal of Ethnopharmacology* and *Nutrients* in the field of TCM treatment for diabetes through gut microbiota.

**TABLE 3:** Top 10 journals with the most published.

Sources	Documents	IF (2023)	Cites
Frontiers in Pharmacology	31	5.6	336
Journal of Ethnopharmacology	31	5.4	779
Food and Function	26	6.1	718
Nutrients	26	5.9	1,000
Frontiers in Microbiology	23	5.2	366
Journal of Functional Foods	18	5.3	485
Frontiers in Endocrinology	16	5.2	183
Biomedicine and Pharmacotherapy	15	7.5	483
Frontiers in Nutrition	15	5	143
Journal of Agricultural and Food Chemistry	14	6.1	9

**TABLE 4:** Top 10 journals with the most cited.

Sources	Cites	IF (2023)	Documents
Nature	1,147	64.8	0.1
PLoS One	1,095	3.7	11
Journal of Agricultural and Food Chemistry	1,087	6.1	14
Nutrients	1,000	5.9	26
Journal of Ethnopharmacology	779	5.4	31
Food and Function	718	6.1	26
Scientific Reports	714	4.6	10
Food Chemistry	678	8.8	4
Diabetes	659	7.7	0.1
Gut	610	24.5	3

### 3.3 Bursts of references

**FIGURE 5:**
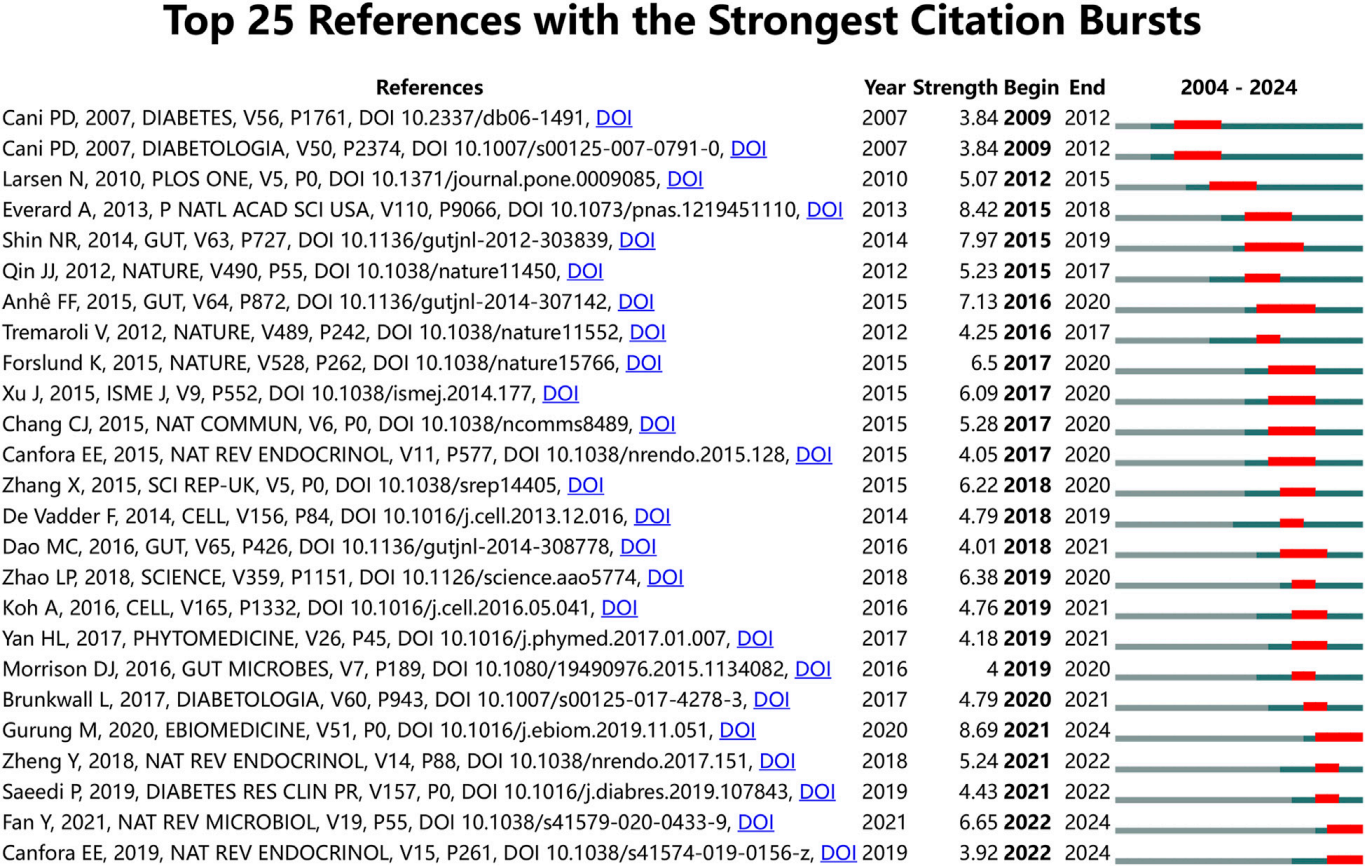
Top 25 References with the strongest citation bursts on TCM regulating gut microbiota in the treatment of diabetes.

To further investigate the forefront and focal areas of TCM treatment for diabetes through gut microbiota, we utilized CiteSpace to identify the top 25 most significant citation bursts (refer to Figure 5). The titles of these citations, along with their respective DOIs, are listed in Supplementary Annex 3. Notably, the three citations exhibiting the most pronounced citation bursts were: 1) “Role of gut microbiota in type 2 diabetes pathophysiology (strength: 8.69)”; 2) “Cross-talk between *Akkermansia muciniphila* and intestinal epithelium controls diet-induced obesity (strength: 8.42)”; 3) “An increase in the Akkermansia spp. population induced by metformin treatment improves glucose homeostasis in diet-induced obese mice (strength: 7.97)”. Furthermore, the titles of the three most cutting-edge citation bursts were: 1) “Gut microbial metabolites in obesity, NAFLD and T2DM”; 2) “Gut microbiota in human metabolic health and disease”; 3) “Global and regional diabetes prevalence estimates for 2019 and projections for 2030 and 2045: Results from the International Diabetes Federation Diabetes Atlas”. Through the reference burst analysis, we have identified three key areas of focus within the field of TCM treatment for diabetes through gut microbiota: 1. The role of gut microbiota in diabetes; 2. The relationship between specific microorganisms and metabolic health; 3. Regulation of gut microbiota by natural products.

### 3.4 Keyword clusters and evolution of themes

Keyword clusters play a pivotal role in swiftly comprehending the principal research themes and trajectories within a specific field. In our investigation, VOSviewer facilitated the identification of 3,691 keywords. Table 5 enumerates the top 20 keywords, each appearing over 40 times, thereby underscoring the key areas of research focus. The keyword “obesity” emerged as the most frequent (n = 207), followed by “insulin resistance” (n = 158), “inflammation” (n = 150), “oxidative stress” (n = 101), “extract” (n = 94), and “metabolism” (n = 78).

**TABLE 5:** The top 20 keywords.

Rank	Keywords	Count
1	Obesity	207
2	Insulin resistance	158
3	Inflammation	150
4	Oxidative stress	101
5	Extract	94
6	Metabolism	78
7	High-fat diet	69
8	Glucose	59
9	Diet	56
10	Mechanism	56
11	*In-vitro*	52
12	Health	49
13	Polysaccharides	49
14	Chain fatty-acids	48
15	Polyphenols	48
16	Association	46
17	Mice	46
18	Antioxidant activity	42
19	Metabolic syndrome	41
20	Double-blind	40

**FIGURE 6:**
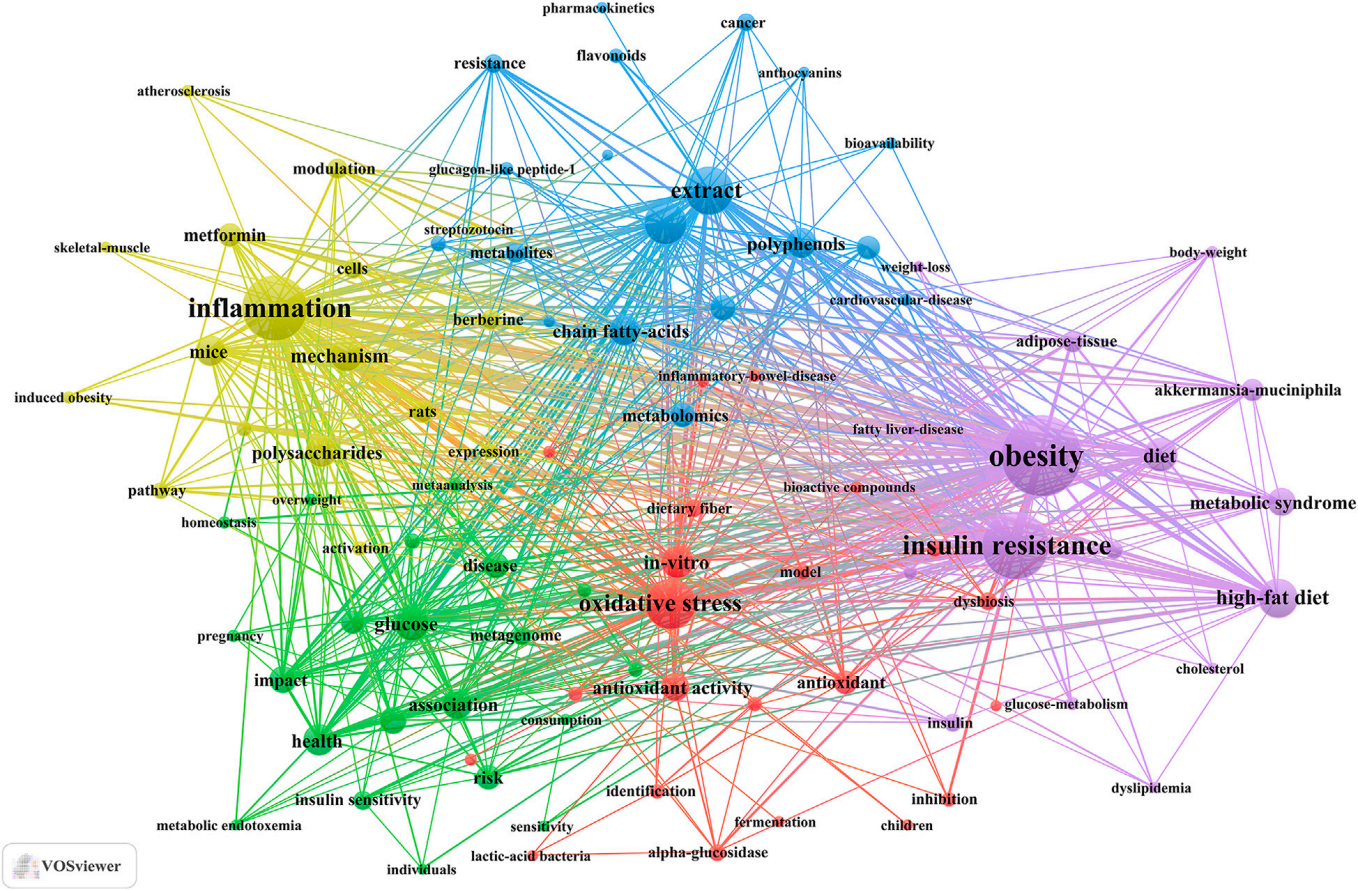
Keywords co-occurrence map of publications on TCM regulating gut microbiota in the treatment of diabetes.

Through cluster analysis, we observe 5 different colored clusters in Figure 6. (1) TCM treats diabetes by regulating gut microbiota and related biological mechanisms (red dots), there are 22 keywords, including alpha-glucosidase, antioxidant, bioactive compounds, butyrate, dietary fiber, and so on. (2) Influence of TCM and diet on diabetes and metabolic health (green dots), there are 21 keywords, including association, consumption, glycemic control, homeostasis, insulin sensitivity, and so on. (3) The role and mechanism of the active components of TCM in the prevention and treatment of metabolic diseases (blue dots), there are 19 keywords, including anthocyanins, flavonoids, polyphenols, metabolism, glucagon-like peptide-1, and so on. (4) Molecular mechanism of TCM and its components in regulation of metabolic diseases (yellow dot), there are 17 keywords, including inflammation, polysaccharides, mechanism, activation, pathway, and so on. (5) The role of TCM and its components in metabolic syndrome and its related metabolic regulation (purple dot), there are 17 keywords, including obesity, metabolic syndrome, lipid-metabolism, glucose-metabolism, cholesterol, and so on. All keywords contained in the four clusters can be found in Supplementary Annex 4.

**FIGURE 7:**
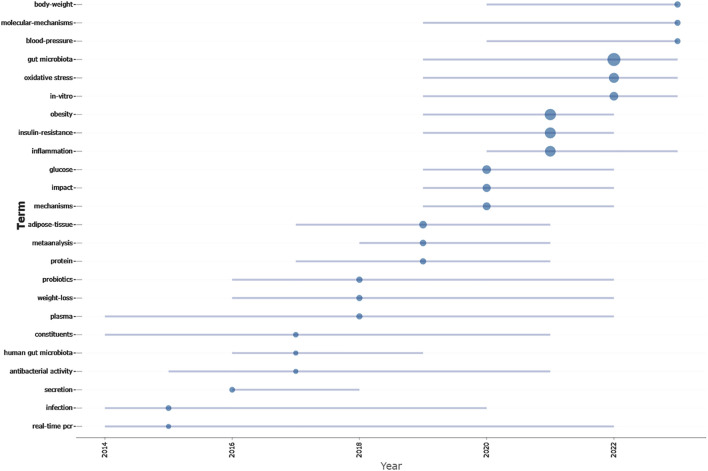
Trend topics on TCM regulating gut microbiota in the treatment of diabetes research.

Moreover, to anticipate forthcoming trends in this domain, we utilized the *Bibliometrix* package within the R software environment to construct a thematic evolution map (Figure 7). Analysis revealed that from 2014 to 2018, this field mainly focuses on the treatment of diabetes by changing gut microbiota of TCM. Transitioning from 2019 to 2023, this field mainly focuses on the molecular mechanism of TCM regulating gut microbiota in the treatment of diabetes.

Overall, through the analysis of keyword clusters and the evolution of themes, we identified two primary research focuses regarding TCM regulating gut microbiota in the treatment of diabetes. (1) The active constituents of TCM, such as flavonoids and polyphenols, in the treatment of diabetes through the modulation of gut microbiota. (2) The mechanisms by which TCM modulates the gut to treat diabetes, including anti-inflammatory and antioxidant effects.

## 4 Discussion

### 4.1 General information

In this study, we compiled an extensive corpus comprising 751 documents spanning the period from 2004 to 2024. The discerned pattern indicates a consistent upward trend in the quantity of documents investigating the interaction between TCM regulating gut microbiota and diabetes, signifying a progressive increase from 2004 to 2022. Surprisingly, the number of publications starts to decrease in 2023 compared to 2022. There are four possible reasons for this result: 1) Complexity of Research: The composition of gut microbiota is highly complex and influenced by individual differences, diet, environment, and other factors (Leshem et al., 2020; Kurilshikov et al., 2021). Establishing a clear cause-and-effect relationship in such a multifaceted context is challenging, and many findings are difficult to replicate or verify. 2) Technical Limitations: Although high-throughput sequencing technology has advanced the study of gut microbiota, analyzing and interpreting the data remains a significant challenge (Gao et al., 2021; Wensel et al., 2022). Current technological methods may not fully capture the dynamic changes and complex interactions within the intestinal flora. 3) Reproducibility of Results: Many early studies reported associations between gut microbiota and various diseases, but subsequent research often found that these results lacked reproducibility, raising concerns about the reliability of the initial studies (Islam et al., 2022). 4) Difficulties in Clinical Application: Despite the theoretical potential of intestinal flora research, translating these findings into practical clinical applications is challenging. Developing treatments based on gut microbiota faces numerous technical and regulatory obstacles. Overcoming these difficulties will help to increase researchers’ interest in the field.

In the domain of research on TCM regulating gut microbiota in the treatment of diabetes, China has emerged as a leading nation, producing the highest volume of scholarly publications. This trend reflects the deep-rooted historical significance of TCM in China, which attracts considerable attention from Chinese researchers. The extensive collection of 751 documents was disseminated across 288 journals, with significant contributions from reputable publications such as *Frontiers in Pharmacology*, *Journal of Ethnopharmacology*, and *Food and Function*. Notably, the *Journal of Ethnopharmacology* distinguished itself as a major focal point, featuring a substantial number of published articles and receiving a considerable volume of citations. This prominence underscores the Journal of Ethnopharmacology as a crucial publication in the field of TCM regulating gut microbiota in the treatment of diabetes, affirming its role as a primary channel for disseminating research findings in this area.

### 4.2 Hotspots and development trends

Through a comprehensive analysis encompassing literature clustering, keyword frequency analysis, keyword clustering, and theme evolution, we have identified the potential research hotspots in the treatment of diabetes through the modulation of gut microbiota by TCM. The findings indicate that the research frontiers and hotspots in this field are primarily concentrated in three areas. Firstly, the active constituents of TCM, such as flavonoids and polyphenols, which treat diabetes by modulating the gut microbiota. Secondly, the mechanisms by which TCM modulates the gut to treat diabetes, including anti-inflammatory and antioxidant effects. Thirdly, the targeting of specific bacterial populations within the gut microbiota by TCM to treat diabetes.

#### 4.2.1 The active constituents of TCM treat diabetes by modulating the gut microbiota

Through our analysis, we found that active constituents of TCM such as flavonoids and polyphenols play significant roles in regulating gut microbiota to treat diabetes. Flavonoid compounds are widely present in many TCM materials, demonstrating various bioactivities in preclinical studies (Almeida et al., 2022; Song et al., 2020; Bharti et al., 2021). The flavonoids can promote the growth of beneficial bacteria like *Bifidobacterium* and *Lactobacillus* (Xiong et al., 2023). These bacteria improve gut barrier function and enhance insulin sensitivity by producing short-chain fatty acids (SCFAs) such as acetate and butyrate (González Hernández et al., 2019). Consequently, they help regulate blood glucose levels. Notably, flavonoids can inhibit harmful bacteria associated with metabolic disorders, such as certain Gram-negative bacteria (Górniak et al., 2019). This inhibition reduces gut inflammation and insulin resistance (Han et al., 2022). These findings offer new insights for the prevention and treatment of diabetes.

Polyphenolic compounds, such as catechins in green tea and proanthocyanidins in grape seeds, also exhibit significant effects in modulating gut microbiota (Wu et al., 2021; Du et al., 2021). Through their potent antioxidant and anti-inflammatory properties, polyphenols reduce oxidative stress and inflammatory responses in the gut, protecting gut barrier function (Truong and Jeong, 2022; Sheng et al., 2020). Specifically, polyphenols can enhance the activity of antioxidant enzymes (e.g., superoxide dismutase and glutathione peroxidase) to scavenge free radicals, reducing oxidative damage to gut cells (Rudrapal et al., 2022). Furthermore, polyphenols can modulate the composition of gut microbiota, increasing the proportion of beneficial bacteria and improving the gut microenvironment, thereby exerting positive effects on diabetes (Li et al., 2018).

It should be noted that there are some shortcomings in the research field regarding the treatment of diabetes through the regulation of gut microbiota by the active ingredients of TCM. (1) Insufficient depth of mechanism studies: Although studies have shown that flavonoids and polyphenols can play an anti-diabetic role by regulating intestinal flora, most of this research has been conducted on animal models or *in vitro* experiments. The specific molecular mechanisms, particularly those in the human body, still need further exploration. (2) Composition complexity: TCM often contain a variety of active ingredients, which may have synergistic or antagonistic effects. Most existing studies focus on the role of single components, with few addressing the interactions among complex components. This gap limits our understanding of the overall efficacy of TCM. To bridge this gap, network pharmacology combined with multi-omics technologies can be employed to construct component-target-pathway network models, providing a more comprehensive understanding of the multi-component, multi-target action mechanisms. Through this approach, key nodes of specific components within the network can be identified, revealing their potential synergistic or antagonistic effects, and predicting the interactions among multiple constituents (Gu et al., 2024; Bi et al., 2024). Additionally, machine learning and artificial intelligence-assisted analyses offer promising tools for exploring potential synergistic patterns and mechanisms from high-dimensional data. In particular, deep learning can help analyze complex datasets and uncover novel interaction patterns among components, providing fresh perspectives on the study of composition complexity (Li et al., 2022). (3) Dose and safety: The effective dose and safety of TCM active ingredients have not been fully defined. In particular, the long-term effects of TCM active ingredients on gut microbiota and overall metabolic health require further verification through clinical studies.

#### 4.2.2 Mechanism of TCM regulating gut microbiota treatment of diabetes

Through the analysis of the literature in this field, we found that anti-inflammatory and antioxidant effects are the most frequently mentioned mechanisms by which TCM regulates gut microbiota in the treatment of diabetes. It is well-established that intestinal inflammation and oxidative stress play key roles in the onset and development of diabetes and its complications (Malik et al., 2020). However, many molecular mechanisms related to the gut microbiome and diabetes have been extensively studied. For example, Sirtuin 1 protects high-fat diet-fed mice from metabolic disorders by modulating *Firmicutes* and *Bacteroidetes* (De Vadder et al., 2014). Mice lacking sirtuin 1 develop adipose tissue hypertrophy, fatty liver, and insulin resistance. Zinc transporter 8 (ZnT8) in pancreatic B-cells is involved in diabetes development. ZnT8 deficiency leads to fat accumulation and glucose intolerance by altering gut microbiota, increasing T2DM and obesity risk (Wan et al., 2017; Mao et al., 2019). Fexaramine, an FXR agonist, improves insulin sensitivity and glucose metabolism by activating the TGR5/GLP-1 pathway and increasing *Acetatifactor* and *Bacteroides* (Pathak et al., 2018). In addition, the intestinal barrier plays a key role, and its dysfunction leads to chronic inflammation (Scheithauer et al., 2020). Hyperglycemia increases intestinal permeability by affecting junction integrity (Thaiss et al., 2018). GLUT2 deficiency maintains barrier integrity, suggesting it as a target for reducing inflammation (Xu et al., 2020). Gut microbiota also affects intestinal permeability via GLP-2 secretion. *Faecalibacterium prausnitzii*’s MAM repairs the intestinal barrier, and *A. muciniphila* improves metabolic health by restoring mucus thickness and enhancing tight junction protein expression (Zhang et al., 2020).

In fact, we have not summarized all the molecular mechanisms related to the gut microbiome and diabetes treatment. But it can be seen that the molecular mechanisms associated with the gut microbiota are definitely not limited to antioxidants and anti-inflammatory. They also include improving insulin resistance (Chen et al., 2021), regulating intestinal barrier function (Chen et al., 2017), influencing the production of SCFAs (Zhao et al., 2020), modulating host immune responses (Zhou et al., 2023), regulating energy metabolism (Xia et al., 2021), inhibiting the proliferation of harmful bacteria, and promoting the colonization of beneficial microbiota (Yang et al., 2021), among various other mechanisms. Therefore, in studies on the mechanism of TCM in regulating gut microbiota treatment of diabetes, researchers should strive to explore a wide range of molecular mechanisms. Overemphasis on anti-inflammatory or antioxidant mechanisms may limit the development of this research field.

In addition, the gut microbiota, as a critical metabolic regulatory center, exhibits complex and multi-layered dynamic interactions in the treatment of diabetes with TCM formulations and antidiabetic drugs. The metabolism of active components in TCM, such as flavonoids, polyphenols, and saponins, relies heavily on enzymatic reactions mediated by gut microbiota (Zhou et al., 2024). These reactions, including hydrolysis, reduction, and demethylation, can convert parent compounds into metabolites with higher bioactivity, thereby enhancing their pharmacological effects (Pant et al., 2023). However, dysbiosis of the gut microbiota may hinder this metabolic transformation, reducing the efficacy of TCM and potentially generating harmful metabolites (Gong et al., 2020). Notably, antidiabetic drugs like metformin alter the composition of the gut microbiota, directly regulating metabolic pathways and indirectly influencing the bioavailability and pharmacological activity of TCM components (Zhang et al., 2021). For example, metformin increases the abundance of short-chain fatty acid-producing bacteria, which improves insulin sensitivity (Mueller et al., 2021). This microbiota change may synergistically enhance the anti-inflammatory and hypoglycemic effects of certain TCM components. However, such complex synergy can be disrupted by competition within metabolic pathways. Specifically, when TCM components and antidiabetic drugs share hepatic enzymes (e.g., the CYP450 family) or intestinal transport proteins, significant pharmacokinetic and pharmacodynamic changes may arise (Xu et al., 2021b). Importantly, drug interactions mediated by gut microbiota involve multiple potential mechanisms, including metabolic synergy, regulation of signaling pathways, and competitive metabolism (Wan and Zuo, 2022; Li et al., 2019). For instance, gut microbiota may produce indole derivatives or secondary bile acids that modulate the pharmacological activity of TCM components by influencing the activation of critical receptors such as FXR and TGR5 (Lin et al., 2021; Xu et al., 2024). Meanwhile, the bioaccumulation of antidiabetic drugs in the gut microbiota warrants close attention. Studies have shown that commonly used antidiabetic agents, such as gliclazide, can undergo bioaccumulation within the microbiota, potentially influencing drug metabolism and pharmacokinetics (Đanić et al., 2023). This may affect the pharmacological activity, therapeutic outcomes, and adverse reactions of TCM. Additionally, interactions between the gut microbiota’s barrier function or immune system may further modulate the pharmacodynamics and pharmacokinetics of these drugs (Tsunoda et al., 2021). A comprehensive understanding of these mechanisms will help elucidate the potential benefits and risks of combining TCM formulations with antidiabetic drugs, thereby providing a theoretical basis for their clinical application.

#### 4.2.3 TCM regulates specific gut bacteria in treating diabetes

Numerous studies have demonstrated that TCM can improve diabetes by modulating specific bacterial populations within the gut microbiota. Wei et al. showed that ginsenoside Rg5 reduces blood glucose levels in diabetic db/db mice. Subsequent research indicated that ginsenoside Rg5 significantly increased the abundance of *Bacteroidetes* and *Proteobacteria*, while decreasing *Firmicutes* and *Verrucomicrobia* in the gut of diabetic db/db mice (Wei et al., 2020). Aloin altered the gut bacterial community by increasing *Bacteroidota* prevalence and reducing the diversity of *Firmicutes*, *Proteobacteria*, and *Actinobacteriota*. Consequently, this intervention mitigated weight loss, lowered fasting blood glucose levels and hemoglobin A1c, enhanced glucose tolerance, and increased fasting serum insulin in T2DM rats (Zhong et al., 2022). Additionally, licorice extract regulates intestinal microbiota balance by increasing *Akkermansia*, *Alloprevotella*, and *Bacteroides* content, while decreasing Lachnospiraceae content, effectively improving diabetes progression (Zhang et al., 2022b).

Despite numerous research achievements in this field, there remain underlying issues that must be addressed. Firstly, whether TCM can accurately and consistently target specific bacterial populations to avoid adverse effects on the overall gut microbiota ecology requires further investigation. Current research primarily focuses on short-term intervention effects, with long-term impacts remaining unclear. Secondly, the gut microbiota is a complex ecosystem where interactions among different microbial groups exist. TCM’s regulation of specific bacterial populations may trigger reactions from other microbial groups, thereby influencing overall therapeutic efficacy. Currently, research on these complex interactions is limited. Furthermore, despite demonstrating potential in regulating gut microbiota in laboratory and animal models, translating TCM into practical clinical therapies faces challenges such as individual differences in drug metabolism and bioavailability.

### 4.3 Limitations

Our study has two main limitations. Firstly, we utilized the WoS database as our sole data source, which may have inadvertently excluded relevant literature available in other databases and potentially led to an overrepresentation of publications from certain countries or regions. Nonetheless, the WoS database is widely regarded as a premier repository for academic works and a highly appropriate platform for bibliometric analysis. Its robust citation network and comprehensive coverage provide a solid foundation for in-depth and reliable analyses, even though it may introduce some degree of regional or journal-specific bias. Furthermore, we performed searches in other databases, such as PubMed and Scopus, using similar search strategies and found that most of the literature overlapped with that in the WoS database. Considering that the WoS database provides the most comprehensive citation data, we chose to rely on a single database to ensure the depth of our data analysis. Even though this approach excluded a small number of documents, it does not compromise the reliability of our analysis. Secondly, we restricted our search to English-language literature, which may introduce bias into our study results by excluding non-English publications. This approach may disproportionately affect research related to TCM, as such studies are often published in local languages or in journals with restrictive publication policies concerning studies on herbal medicine or local plant extracts. Nevertheless, it is noteworthy that English is one of the most widely used languages globally, and English literature generally provides extensive coverage of relevant content. Despite these limitations, our study remains robust and reliable.

## 5 Conclusion

In summary, our study identifies three research hotspots in this field: the role of active constituents of TCM in treating diabetes through modulation of the gut microbiota, mechanisms by which TCM regulates the gut microbiota in diabetes treatment, and TCM regulates specific gut bacteria in treating diabetes. Furthermore, while TCM shows immense potential in treating diabetes through gut microbiota modulation, several shortcomings remain in understanding mechanisms, individual differences, long-term effects, and clinical applications. Future research should address these gaps by further exploring the specific mechanisms of action of active ingredients in TCM, assessing their efficacy and safety in diverse populations, and developing more precise and effective treatment strategies.

## Data Availability

Publicly available datasets were analyzed in this study. This data can be found here: web of science database.
