# Supplementary material for: Global bibliometric analysis of traditional Chinese medicine regulating gut microbiota in the treatment of diabetes from 2004 to 2024
Source: Front Pharmacol. 2025 Jan 23;16:1533984. doi: 10.3389/fphar.2025.1533984 (PMC11799270; doi:10.3389/fphar.2025.1533984)
Supplement: Supplementary file 3 [file DataSheet1.docx]

**Description of Vosviewer software parameters:**

These parameters were selected to present visually appealing and appropriate data figures without impacting subsequent analyses. In the actual analysis, we consider all data and are not limited to those displayed in the figures. For instance, when setting the "minimum occurrence of a specific keyword ≥10," our goal was to display fewer than 200 keywords to avoid overcrowding the figure while still showing as much data as possible without altering the analysis outcomes. The complete dataset used for the analysis is available in the Annexes 2-4.

**Description of R Software Usage**

In this study, the *Bibliometrix* and *ggplot2* packages were utilized. The *Bibliometrix* package, once installed and loaded in R, allows users to enter the command `biblioshiny()` to launch a web-based interface. Data can then be analyzed directly through the user-friendly prompts on the webpage, eliminating the need for additional coding.

The *ggplot2* package was employed to create Figure 3. The code used for this purpose is as follows:

p1 = read.csv("Most Relevant Sources.csv",header=TRUE,check.names = FALSE)

p1$Sources=factor(p1$Sources,levels=p1$Sources)

P2<-ggplot(p1,aes(IF,Sources))+geom_point(aes(size= Cites,color=Articles))+scale_color_gradient(low="Blue",high ="red" )+

scale_size_continuous(range = c(3, 10))+

labs(size="Cites",color=expression(Articles), x="IF", y="Sources",title="Most Relevant Sources")+ theme_bw()

P2+ggtitle("Most Relevant Sources")+theme(plot.title = element_text(hjust = 0.5,size = rel(1.5)))+

theme(axis.text.x = element_text(size = rel(1.5)))+theme(axis.text.y = element_text(size = rel(1.5)))+

theme(axis.title.x = element_text(size =15))+theme(axis.title.y = element_text(size = 3))+

coord_fixed(ratio = 150/10)+scale_y_discrete(limits=rev(levels(p1$Sources)))+theme(text=element_text(family = "serif"))

**Description of Citespace Software Usage**

In this study, the Citespace software was used for bibliometric analysis. For the Time Slicing parameter, we selected "1" for Year Per Slice. All other parameters were kept at their default settings.
